# Evaluation of diagnostic factors used to refer children with constipation for rectal biopsies

**DOI:** 10.1007/s00384-021-04069-4

**Published:** 2021-12-09

**Authors:** Emilie G. Jaroy, Ragnhild Emblem, Henrik M. Reims, The Tien Mai, Gabriel T. Risa, Rune Ougland

**Affiliations:** 1 Department of Pediatric Surgery, Oslo University Hospital, 0424 Rikshospitalet, Norway; 2 Department of Microbiology, Oslo University Hospital, 0372 Rikshospitalet, Norway; 3 Faculty of Medicine, Institute of Clinical Medicine, University of Oslo, 0372 Oslo, Norway; 4 Department of Pathology, Oslo University Hospital, 0424 Rikshospitalet, Norway; 5 Oslo Centre for Biostatistics and Epidemiology, Department of Biostatistics, University of Oslo, 0372 Oslo, Norway; 6 MRC-Laboratory for Molecular Cell Biology, University College London (UCL), London, UK

**Keywords:** Rectal biopsy, Hirschsprung’s disease, Constipation in children, Rome 4 criteria for functional constipation, Pediatric surgery

## Abstract

**Purpose:**

Children with constipation and suspected Hirschsprung’s disease are referred for rectal biopsy. Since this is an invasive procedure, appropriate indications should be applied to minimize the number of “unnecessary” biopsies.

**Methods:**

We reviewed all constipated children who underwent a rectal biopsy to diagnose a possible Hirschsprung’s disease at a tertiary referral hospital over a 6-year period (2013–2018). We registered clinical and demographic factors in these children and conducted correlation and multivariate regression analysis to evaluate the relation between these factors and a diagnosis of Hirschsprung’s disease.

**Results:**

We identified 225 children, aged 0–17 years. In total, Hirschsprung’s disease was diagnosed in only 49/225 (22%). Among the 49 children with Hirschsprung’s disease, 29 (59%) were diagnosed in the neonatal period. Among girls, HD was confirmed in only 10/101 (10%) children, and only 1 of these 10 girls was older than 6 months at the time of the biopsy. The following factors correlated significantly with Hirschsprung’s disease diagnosis in children older than 1 month: “male sex”, “failure to thrive”, “gross abdominal distention plus vomiting” and “fulfils the Rome 4 criteria for functional constipation”.

**Conclusion:**

In children referred for rectal biopsy, the factors most indicative of Hirschsprung’s disease were “male sex”, “failure to thrive”, “gross abdominal distention plus vomiting” and “fulfils the Rome 4 criteria for functional constipation”. Notably, the prevalence of Hirschsprung’s disease decreased with the increasing age of the children. Girls referred for a biopsy rarely had Hirschsprung’s disease, especially those older than 1 month.

**Supplementary information:**

The online version contains supplementary material available at 10.1007/s00384-021-04069-4.

## Introduction

Children with constipation are referred for a rectal biopsy when a suspicion of Hirschsprung’s disease (HD) has been raised. To diagnose HD, rectal biopsies are examined for the presence of ganglion cells. The biopsies are classified as positive for HD (aganglionic) if no ganglion cells are detected after examination of a sufficient number of histological Sections. [1], often supplemented with immunohistochemical staining and/or enzyme histochemistry [2].

**Fig. 1:**
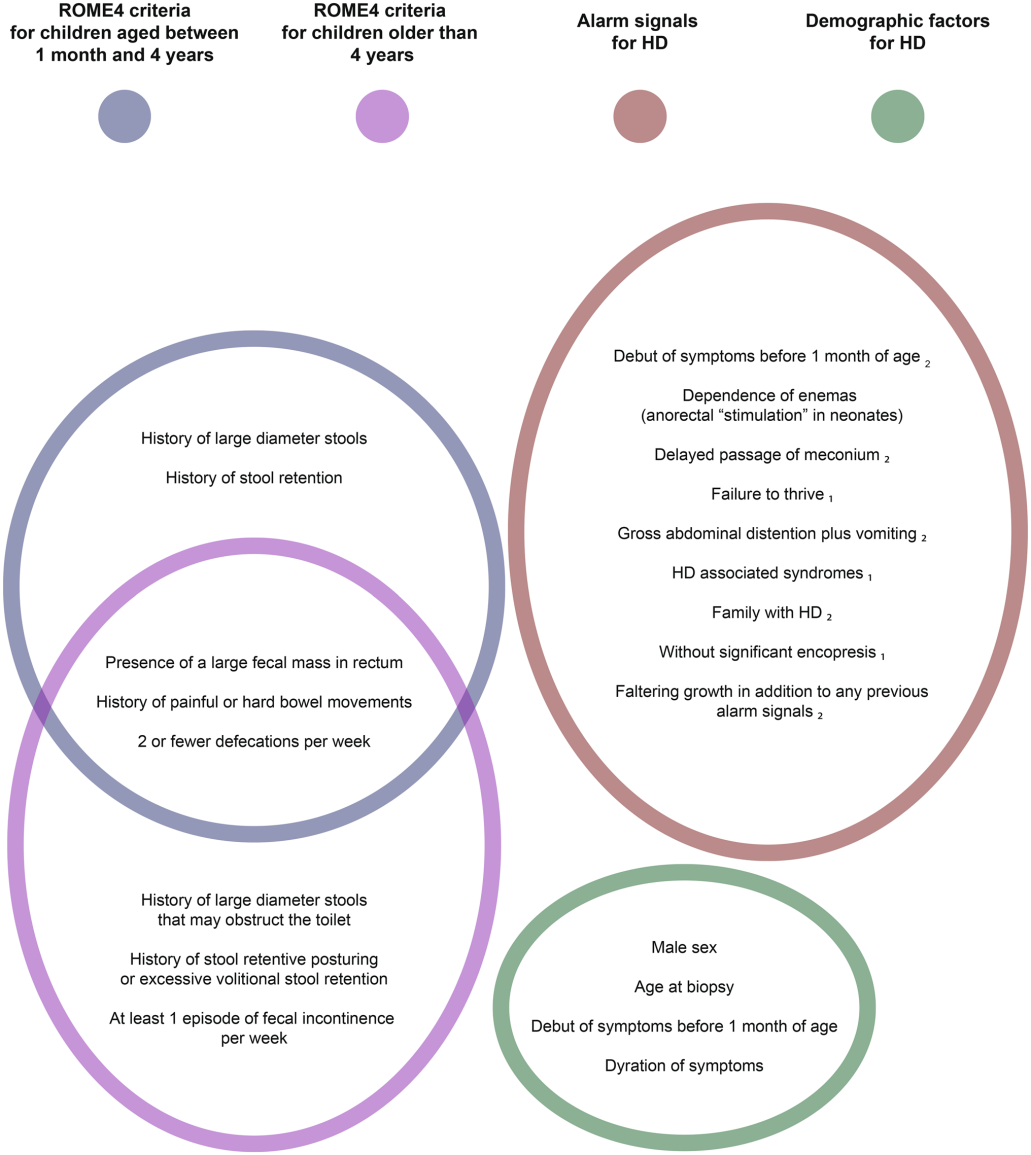
Overview of registered factors: Rome 4 criteria (separate criteria for children aged 1 month to 4 years and children older than 4 years), demographic factors and alarm signals for HD. The listed alarm signals were suggested by ^1^ Langer [7]^2−^ Amiel et al. [9] ^3^ NICE-guidelines [6] and applies only to children older than 4 years

We have observed that relatively few of the rectal biopsies taken at our institution are aganglionic. Thus, we and others suspect that too many biopsies are performed [3]. In line with this, an HD incidence of only 19% was found in a systematic review that included 58 studies with a total of 14,053 rectal suction biopsies [4]. This statistic reflects how challenging the clinical evaluation of constipation is, but also the fear of missing this diagnosis in a constipated child. Constipation is a very common condition with a plethora of causes, and in the vast majority of children, no underlying medical condition is found [5]. To standardize the management of children presenting with constipation, a set of evidence-based guidelines have been developed [5–9]. According to these guidelines, accepted indications for rectal biopsies in children after the neonatal period is constipation according to the Rome 4 criteria for functional constipation (FC) in addition to one or more specified alarm signals (Fig. 1). We registered the relevant clinical and demographic factors in children who underwent rectal biopsy at the Oslo University Hospital. Then, by comparing children diagnosed with and without HD, we sought to evaluate which of these factors best indicate rectal biopsy.

## Patients and methods

By mining electronic clinical records, we did a retrospective evaluation of patients aged 0–17 years undergoing rectal biopsies in the period 2013–2018 at the Department of Gastrointestinal and Pediatric Surgery at Oslo University Hospital, Norway. Pediatricians evaluated the constipated children and referred them for rectal biopsy, and the pediatric surgeons performed the procedure.

### Inclusion criteria and division into age groups

Two hundred twenty-five patients with a conclusive biopsy documented in their clinical record were identified and included in the study. We stratified the children into (i) neonates and (ii) children older than 1 month. Where relevant, we divided the children older than 1 month into the following two subgroups: (i) children aged 1 month–4 years and (ii) children older than 4 years. A total of 47 neonates and 178 children older than 1 month were included in the study (111 children aged 1 month–4 years and 67 children older than 4 years).

### Clinical data

We registered demographic factors, conservative treatment before referral, the Rome 4 criteria for FC and alarm signals for HD (Fig. 1). Diagnostic criteria for FC, according to the Rome 4 criteria, must include 2 or more clinical factors occurring at least once per week for a minimum of 1 month, along with insufficient criteria for a diagnosis of irritable bowel syndrome. The list of factors differs slightly for children younger and older than 4 years (Fig. 1) [6]. Notably, the Rome 4 criteria do not apply to neonates, because symptoms are required to have persisted for at least 1 month. Furthermore, the factor “failure to thrive” was assigned to a child if their weight for age falls below the 5th percentile or crosses two major percentile lines on a growth chart [10].

In neonates, we only registered “sex”, “delayed meconium” (after 48 h) and “use of anorectal stimulation”. We interpreted the alarm signal “dependence of enemas” regarding neonates as anorectal stimulation [7]. In children older than 4 years, we also registered “fecal incontinence/encopresis”. Both the terms “fecal incontinence” and “encopresis” have previously been used to describe involuntary defecation into the child’s underwear after the age of 4 years. Presently, the term “encopresis” has been replaced by “fecal incontinence”, which is now defined as a term with no distinction made on the basis of presumed etiology [11, 12]. Therefore, to avoid the use of two terms, we will refer to the alarm signal “without significant encopresis” suggested by Langer [7] as “without significant fecal incontinence”. We did not include results from anorectal manometry and contrast enemas because of limited data and because we found it difficult to interpret and categorize the results from these examinations. The HD-associated syndromes included in our study were trisomy 21 (Down’s syndrome), Waardenburg-Shah, Currarino and Congenital central hypoventilation syndrome.

### Ethics

The study was approved by the Oslo University Hospital’s Commission for Personal Security, no 18/21406.

### Biopsy technique

Rectal suction biopsies (RSB) or full-thickness biopsies (FTB) were taken 2–4 cm above the dentate line on the posterior rectal wall. When RSBs were used, 2–3 biopsies were taken simultaneously, using rbi2 rectal biopsy system manufactured by Aus Systems. RSBs are commonly favoured in neonates and younger infants and FTBs in older children [13, 14].

### Biopsy evaluation

RSBs were classified as ganglionic when one or more ganglion cells were identified in the submucosa and FTBs when ganglionic cells were identified in both the submucosa and the myenteric plexus. Biopsies were considered aganglionic if no ganglion cells were identified after examination of ≥ 50 representative sections stained with haematoxylin and eosin.

### Statistical analysis

All factors and patient groups queried in relation to HD were first subjected to Pearson correlation analyses and then followed up with multivariate logistic regression analyses. Only factors with a correlation value exceeding ± 0.15 and a *p* value below 0.05 were subjected to multivariate logistic regression analysis. In the specific cases where the multivariate logistic regression analyses ran into the zero-cell problem, a Haldane-Anscombe correction was applied, as advised by Weber and Knapp [15–17]. Perfect separation precluded multivariate logistic regression analyses of the smaller datasets with children above the age of 4.

Significance was defined as *p* < 0.05 and *p* values below 0.01 were marked as “ < 0.01”. In tables, median values are reported with lowest-highest values in parenthesis. Throughout the study, numbers were rounded to 2 decimal places and percentages were presented without decimals.

## Results

**Fig. 2:**
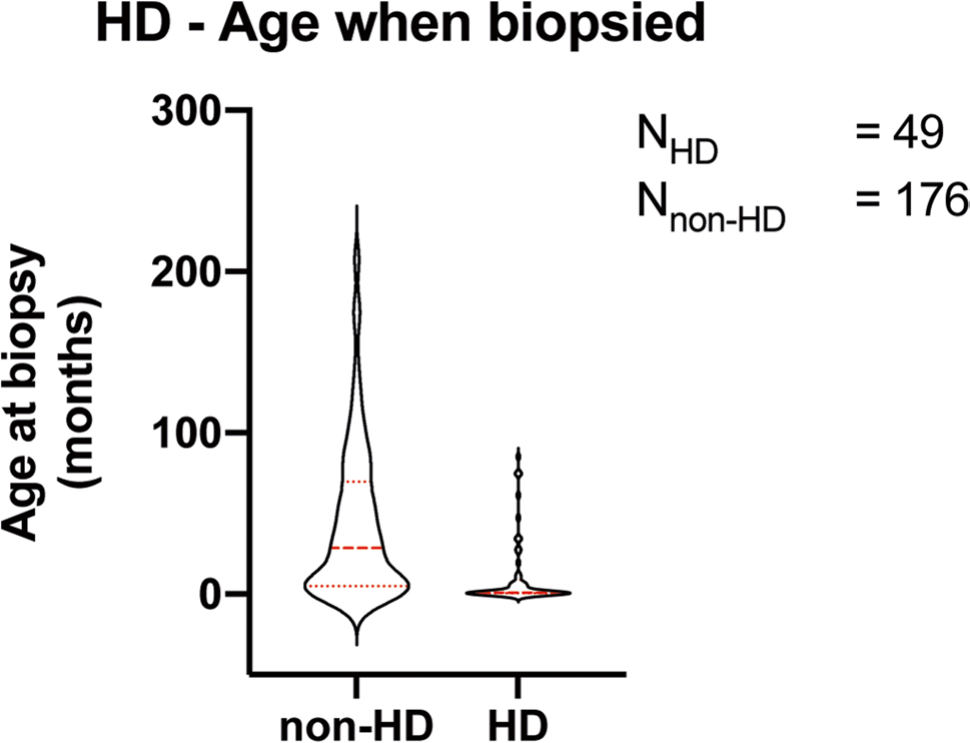
Age when biopsied for children with and without a resulting HD diagnosis. The mean age is visualized by a thick red dotted line and the 25% and 75% quartiles by thin red dotted lines

**Fig. 3:**
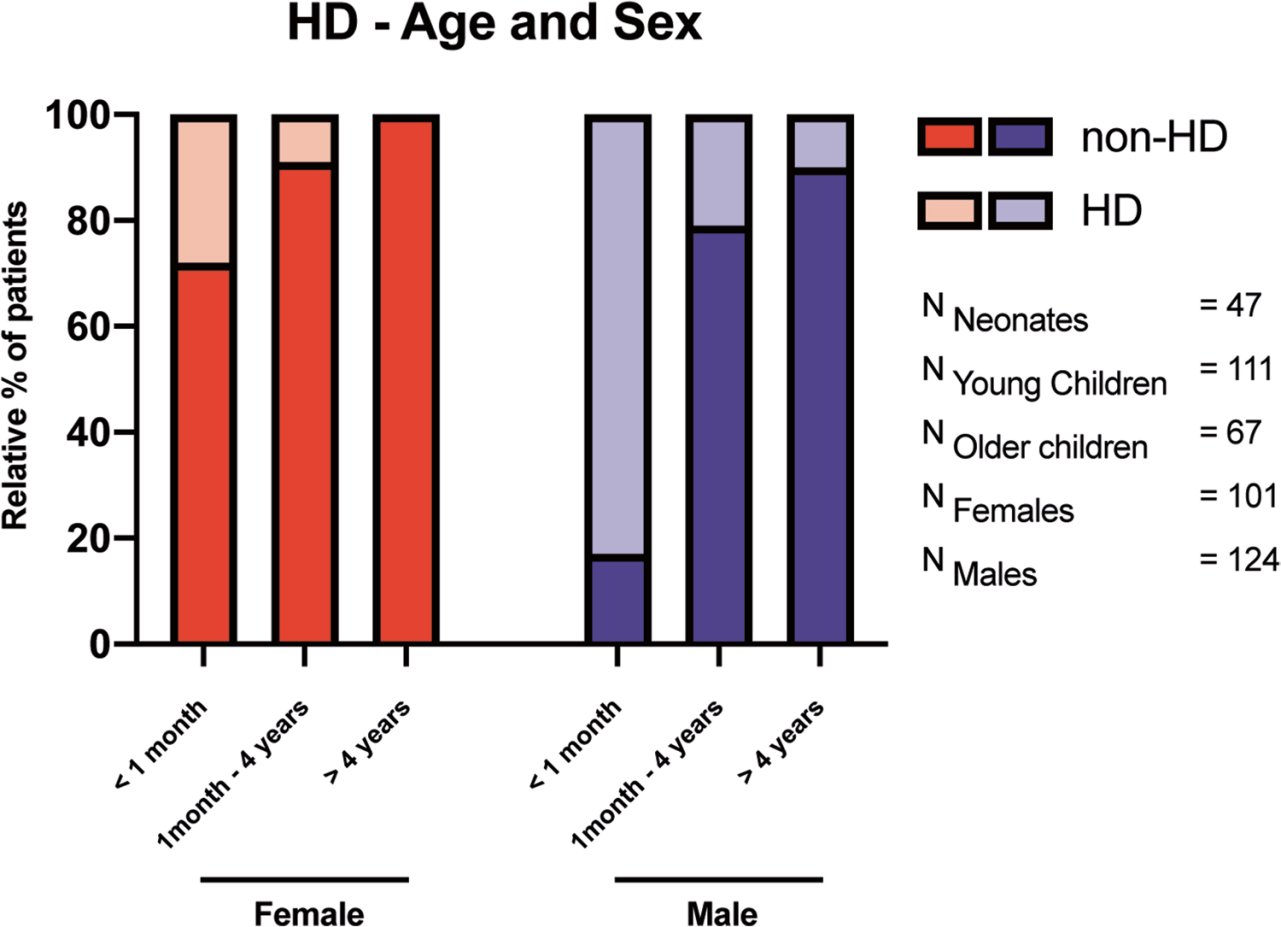
The prevalence of HD sorted by age and sex. Notably, girls older than 1 month rarely had HD

From 2013 to 2018, 225 children with constipation, aged 1 day–17 years, had a rectal biopsy at the Oslo University Hospital. Of these children, 49/225 (22%) were diagnosed with HD, among whom 29 (59%) were diagnosed in the neonatal period. In general, children with HD were younger at the time of biopsy than children without HD (Fig. 2 and Table 4). The prevalence of HD and sex in different age groups are given in Table 1 and Fig. 3. In total, almost as many girls as boys had a rectal biopsy taken (101 girls vs. 124 boys). However, boys were about 4 times more likely to have HD (39 boys/10 girls). Among the 10 girls with HD, only 1 was older than 6 months when the diagnosis was made. Notably, only 4 children were diagnosed with HD after 4 years of age, all of whom were male.

**Table 1:** Prevalence of HD relative to sex (including all 225 children). The children were stratified into (i) neonates and (ii) children older than one month. The children older than 1 month were further separated into the following two subgroups: (i) children aged 1 month to 4 years and (ii) children older than 4 years

Age	Total	Female	Male
Neonates (< 1 month)	47 (29 HD ≈ 62%)	18 (5 HD ≈ 28%)	29 (24 HD ≈ 83%)
> 1 month	178 (20 HD ≈ 11%)	83 (5 HD ≈ 6%)	95 (15 HD ≈ 16%)
1 month–4 years	111 (16 HD ≈ 14%)	58 (5 HD ≈ 9%)	53 (11 HD ≈ 21%)
> 4 years	67 (4 HD ≈ 6%)	25 (0 HD = 0%)	42 (4 HD ≈ 10%)

## Clinical data

### Neonates

The study included 47 neonates (median age 10 days), and more than half had positive biopsies. Moreover, 83% of the neonates diagnosed with HD were boys (Table 1). There was no significant difference regarding “anorectal stimulation” or “delayed meconium” among neonates with and without HD. The only significant factor indicative of HD was “male sex” (Tables 2 and 3).

**Table 2:** Factors relevant for diagnosing HD in neonates (47 children). Correlation analysis

Factor category	Factor	Non-HD [18]	HD [29]	Correlation with HD	Confidence interval	*p* value
Demographic factor	Male sex	5/18 (≈ 28%)	24/29 (≈ 83%)	0.55	0.32–0.72	< 0.01
Alarm signal for HD	Delayed meconium	11/18 (≈ 61%)	19/29 (≈ 65%)	0.05	−0.25 to 0.33	0.77
Alarm signal for HD	Anorectal simulation	1/18 (≈ 6%)	4/29 (≈ 14%)	0.13	−0.16 to 0.40	0.38

**Table 3:** Factors relevant for diagnosing HD in neonates (47 children). Multivariate logistic regression analysis

Factor category	Factor	Odds	Standard error	95% CI (profile likelihood)	*p* value
	Intercept	0.38	0.53	−2.09 to 0.02	0.07
Demographic factor	Male sex	12.48	0.72	1.19–4.04	< 0.01

### Children older than 1 month

This age group included 178 children, aged 1 month–17 years (median age 32 months). The median age at biopsy was lower in children with than without HD (15.5 vs. 35 months). Twenty of 178 (11%) had a positive biopsy for HD, 15/20 (75%) were boys and 5/20 (25%) were girls. Four of these 5 girls with HD were aged 4–6 months when the rectal biopsy was taken, and the fifth girl was 34 months old (Table 1).

All children with HD fulfilled the Rome 4 criteria for FC, compared to 63% of children without HD, among which the criterion “2 or fewer defecations per week” differed significantly between the group with and without HD (Table 4 and S4). Furthermore, we found that “gross abdominal distention plus vomiting” occurred in 65% (13/20) of the children with HD vs. 4% (7/158) of children without HD, “failure to thrive” in 70% (14/20) vs. 24% (38/158) and “debut of symptoms before 1 month” in 65% (13/20) vs. 56% (88/158). We found no significant difference in the prevalence of “HD-associated syndromes” between children with HD and without HD (10% vs. 8%), and only two children (one with and one without HD) had “family with HD” documented in their clinical records. Moreover, among the children without HD, 21% (33/158) also had “delayed meconium”. Differences in factors between patients with and without HD are listed in Table 4.

**Table 4:** Factors relevant for diagnosing HD in children older than 1 month (178 children). Correlation analysis, Haldane-Anscombe corrected

Factor category	Factor	Non-HD (158)	HD [20]	Correlation with HD	Confidence interval	*p* value
ROME 4 criteria for functional constipation	ROME4 ( +)	99/158 (≈ 63%)	20/20 (= 100%)	0.25	0.11–0.38	< 0.01
Demographic factor	Male sex	80/158 (≈ 51%)	15/20 (≈ 75%)	15.00	0.01–0.29	**0.04**
Demographic factor	Age at biopsy (months)	35 (1–209)	15.5 (2–85)	−0.15	−0.29 to −0.00	**0.048**
Demographic factor	Debut of symptoms before 1 month of age	88/158 (≈ 56%)	13/20 (≈ 65%)	0.06	−0.09 to 0.20	0.43
Demographic factor	Duration of symptoms (months)	31 (0.13–207)	22.5 (1–74)	−0.13	−0.15 to 0.15	0.09
Alarm signal for HD	Dependence of enemas	62/158 (≈ 39%)	14/20 (≈ 70%)	0.20	0.05–0.33	**0.01**
Alarm signal for HD	Delayed meconium	33/158 (≈ 21%)	9/20 (≈ 45%	0.18	0.03–0.32	**0.02**
Alarm signal for HD	Failure to thrive	38/158 (≈ 24%)	14/20 (≈ 70%)	0.32	0.18–0.45	** < 0.01**
Alarm signal for HD	Gross abdominal distention plus vomiting	7/158 (≈ 4%)	13/20 (≈ 65%)	0.61	0.50–0.69	** < 0.01**
Alarm signal for HD	HD-associated syndromes	12/158 (≈ 8%)	2/20 (≈ 10%)	0.03	−0.12 to 0.17	0.71
Alarm signal for HD	Faltering growth in addition to any previous alarm signal	20/158 (≈ 13%)	2/20 (≈ 10%)	−0.03	−0.17 to 0.12	0.23

The following factors were significantly more common in children with than without HD: “male sex”, “failure to thrive” and “gross abdominal distention plus vomiting”. In addition, the factor “fulfils the Rome 4 criteria for FC” differed between the groups at a *p* value of 0.05 (Table 5). The following alarm signals did not differ significantly: “Dependence of enemas” and “delayed meconium”.

**Table 5:** Factors relevant for diagnosing HD in children older than 1 month (178 children)..Multivariate logistic regression analysis, Haldane-Anscombe corrected

Factor category	Factor	Odds	Standard error	95% CI (profile likelihood)	*p* value
	Intercept	0.0004152	1.971	−13.27 to − 4.70	< 0.01
ROME 4 criteria for functional constipation	ROME4 ( +)	24.18	1.637	0.76–4.87	0.05
Demographic factor	Male sex	5.676	1.736	0.31–3.42	**0.03**
Demographic factor	Age at biopsy	0.9844	0.01279	−0.04 to 0.01	0.22
Alarm signal for HD	Dependence of enemas	2.575	0.7898	−0.57 to 2.60	0.23
Alarm signal for HD	Delayed meconium	2.058	0.8114	−0.93 to 2.33	0.37
Alarm signal for HD	Failure to thrive	5.635	0.7992	0.36–3.29	**0.02**
Alarm signal for HD	Gross abdominal distention plus vomiting	24.57	0.7737	1.78–4.87	** < 0.01**

Importantly, good response to conservative treatment may eliminate the indication for rectal biopsy. We found that nearly all (176/178) of the children older than 1 month had documented conservative treatment before referral and that they were referred for rectal biopsy because they did not have a satisfactory response. We also compared what kind of conservative treatment the children with and without HD had; 55% vs. 80% had laxatives, 20% vs 23% had dietary advice, 30% vs.27% needed rectal stimulus for defecation, 5% vs 12% had laxoberal droplets and 70% vs 39% were dependent on enemas.

Focusing separately on the 67 children older than 4 years (median age 83 months), only 6% (4/67) had a positive biopsy and all were boys (Fig. 3). Moreover, all 4 boys with HD fulfilled the Rome 4 criteria, and all reported to have one or more of the alarm signals. Notably, 3 of the 4 patients older than 4 years with HD experienced fecal incontinence. The only factors which significantly correlated with HD were “failure to thrive” and “gross abdominal distention plus vomiting” (Table S3a).

## Discussion

Children with constipation are referred for rectal biopsy when a suspicion of HD has been raised. As a rectal biopsy is an invasive procedure with possible complications [18–20], the indications for a biopsy should be as accurate as possible, to avoid taking too many negative biopsies and missing positive cases. However, defining the indications for rectal biopsies remains challenging despite available guidelines [5–9]. In the present study, we assessed the most appropriate indicative factors for referring children for rectal biopsy. “Male sex” was the only factor that differed significantly between children with and without HD in the neonatal group. Among children older than 1 month, “failure to thrive” and “gross abdominal distention plus vomiting” were indicative of HD, in addition to “male sex”. Also, “fulfils the Rome 4 criteria for FC” was significantly correlated to HD diagnosis, among which the criterion “2 or fewer defecations per week” was particularly important.

Our study confirms “male sex” as an important indicative factor for HD in a constipated child. In accordance with this, we found that girls referred for a biopsy rarely had HD, especially if they were older than 1 month. This finding is consistent with data from Muise et al., who reported a low prevalence of HD in girls compared to boys (16% vs 61%) [13]. Among HD patients we found a male-to-female ratio of 4:1, when including all age groups. This confirms the male predominance found in earlier studies [9, 21–23]. In view of the much higher prevalence of HD among males than among females, we find it concerning that almost as many girls as boys were referred for rectal biopsy. Although the fear of missing a HD diagnosis is great, the secure knowledge of the large gender difference could hopefully result in a more deliberate referral practice for girls.

Furthermore, our study contrasts with the traditional teaching that the vast majority of children with HD present during the neonatal period with neonatal obstruction or enterocolitis [7]. While Singh et al. [24] found that 90% (114/126) of children with HD were diagnosed in the neonatal period, we found that only 59% of the children with HD were diagnosed in the neonatal period. In agreement with our results, Rahman et al. [25] found that 53% of positive diagnoses were made in children presenting after the neonatal period. It is unclear why as many as 41% of the HD patients in our study were diagnosed after the neonatal period. One possible explanation is that Norwegian neonates are normally sent home from the maternity ward only 2 days after birth, leaving little time for observation.

Regarding “gross abdominal distention plus vomiting”, our findings are coherent with previously published data [8]. In a study including both neonates and older children, Philips et al. found that a significantly higher percentage of the children with HD presented with abdominal distension and vomiting than children without HD (64 vs 31%). Interestingly, they also found this alarm signal to be the single most important out of the five alarm signals recommended by the NICE guidelines [26]. These findings agree closely with our results. Our results are also in agreement with previously published data regarding the alarm signal “failure to thrive”. Noviello et al. found that three of 31 children older than 1 year who had a rectal biopsy taken were diagnosed with HD and that all these three presented with severe constipation and failure to thrive [27]. Thus, our findings support the inclusion of “gross abdominal distention plus vomiting” and “failure to thrive” on the list of recommended alarm signals.

“Delayed passage of meconium” (after 48 h) is listed as a classic sign suggestive of HD [28]. In a study by Sherry et al. [29], the first passage of meconium occurred within 48 h in 99.8% of 500 normal full-term newborns. In comparison, the first passage of meconium occurred within 48 h in only 39% of neonates without HD in our study. We believe our results regarding neonates differ from studies of reference populations because also the children without HD in our study are children with severe problems with constipation. Looking at the children older than 1 month, our results are in agreement with previously published data; Jung showed that approximately half of children with HD had delayed meconium [30]. Notably, we did not expect as many as 21% of the children older than 1 month without HD to have delayed meconium. We interpret our present findings to suggest that “delayed meconium” does not differ between patients with and without HD in a group of constipated children referred for a rectal biopsy.

Our study confirms the importance of “fulfils the Rome 4 criteria for FC” when referring for rectal biopsy in the suspicion of HD. The prevalence of FC according to these criteria was substantial in the children included in our study, more so in children with HD (100%) than without HD (63%). In comparison, the prevalence of FC by these criteria was 12% in a normal child population [31, 32]. We have not found any previous reports of the number of children fulfilling the Rome 4 criteria for FC when referred for rectal biopsy. Philips et al. compared children who were indicated to have HD according to the NICE guidelines with children who were not and found that 16% of indicated biopsies were positive for HD, versus 8% of unindicated biopsies [26]. These findings show that following the recommended guidelines for referral for rectal biopsy increases the positive biopsy rate.

Our results suggest that “dependence of enemas” is less important than other alarm signals for HD. We have not found previous studies that compare “dependence of enemas” in children with and without HD. Based on our data we suggest this alarm signal should be considered less relevant than previously thought [7].

Our study suggests that “without significant fecal incontinence” is a poor indicative factor for HD. Thus, our findings are not in agreement with the previous studies of those who have found “without significant fecal incontinence” to be a good indicative factor for HD [7, 33]. In contrast, we found that the presence of fecal incontinence cannot exclude HD as a diagnosis, as 3 of the 4 patients older than 4 years with HD had fecal incontinences. Our findings are in accordance with Pini-Prato et al., who reported that some children with HD do have fecal incontinence but that this occurs in significantly more children with FC (4% vs. 46%) [34]. Therefore, we question whether the factor “without significant fecal incontinence” should remain on the list of recommended alarm signals for HD. Subsequently, we support the listing of “at least one episode of fecal incontinence per week” in the Rome 4 criteria for FC.

Regarding the factors “family with HD” and “HD-associated syndromes”, our findings adhere well with the report from Singh et al. which states that the incidence of associated anomalies in patients with HD ranges from 11 to 30% [24], Down’s syndrome being the commonest [35]. The genetic background of HD is complex, yet we know that the probability of having HD is greater if the child has an additional diagnosis associated with HD and that the disease runs in some families [9]. Thus, genetics is relevant when evaluating the referral for rectal biopsy [28, 36].

When considering referral for rectal biopsy, one faces a dilemma with the competing interests of not missing a diagnosis of HD and minimizing procedure-related adverse consequences for these children. Pediatric surgeons and pediatricians have discussed whether or not it is worth taking this many rectal biopsies. It has been argued that taking many biopsies is justified because of the risk of missing a diagnosis of HD [25, 26] and because of few and low-grade complications [37]. However, we are inclined to agree with Stewart et al. that too many biopsies are taken [3], and we hope that this manuscript can add to the discussion about how to better identify the children in need of a rectal biopsy.

HD is a rare disease (1/5000) [31] and most studies on this patient group are based on a small number of patients. Compared to previous studies, the number of patients referred for biopsy and the number of patients with HD in our study is reasonable, but still quite small. Our data should therefore be interpreted with some caution. Moreover, because of the retrospective nature of this study, there is a potential recall bias and there may also be a lack of documentation regarding some of the factors in the clinical records.

## Conclusions

In this 6-year retrospective study, HD was diagnosed in 22% of all children, and only 10% of all girls referred for rectal biopsy. In children older than 1 month, the factors most strongly related to HD were “male sex”, “failure to thrive” and “gross abdominal distention plus vomiting”. Our study also confirmed the importance of fulfilling the Rome 4 criteria for FC, in particular, the criterion “2 or fewer defecations per week”. Our study revealed no significant difference between HD and non-HD children regarding “dependence of enemas” (“anorectal stimulation” in neonates), “delayed meconium” or “absence of significant fecal incontinence”. We suggest that the threshold should be higher when considering referral of girls, especially after infancy, for rectal biopsy due to suspected HD.

## Supplementary information

Below is the link to the electronic supplementary material.

Supplementary file1 (TIFF 21344 KB)

Supplementary file2 (TIF 35898 KB)

Supplementary file3 (TIFF 5773 KB)

Supplementary file4 (TIFF 14187 KB)

Supplementary file5 (TIFF 6075 KB)

Supplementary file6 (PDF 84 KB)
