# Supplementary figures and images for: Evaluation of diagnostic factors used to refer children with constipation for rectal biopsies
Source: Int J Colorectal Dis. 2021 Dec 9;37(3):597–605. doi: 10.1007/s00384-021-04069-4 (PMC8885502; doi:10.1007/s00384-021-04069-4)

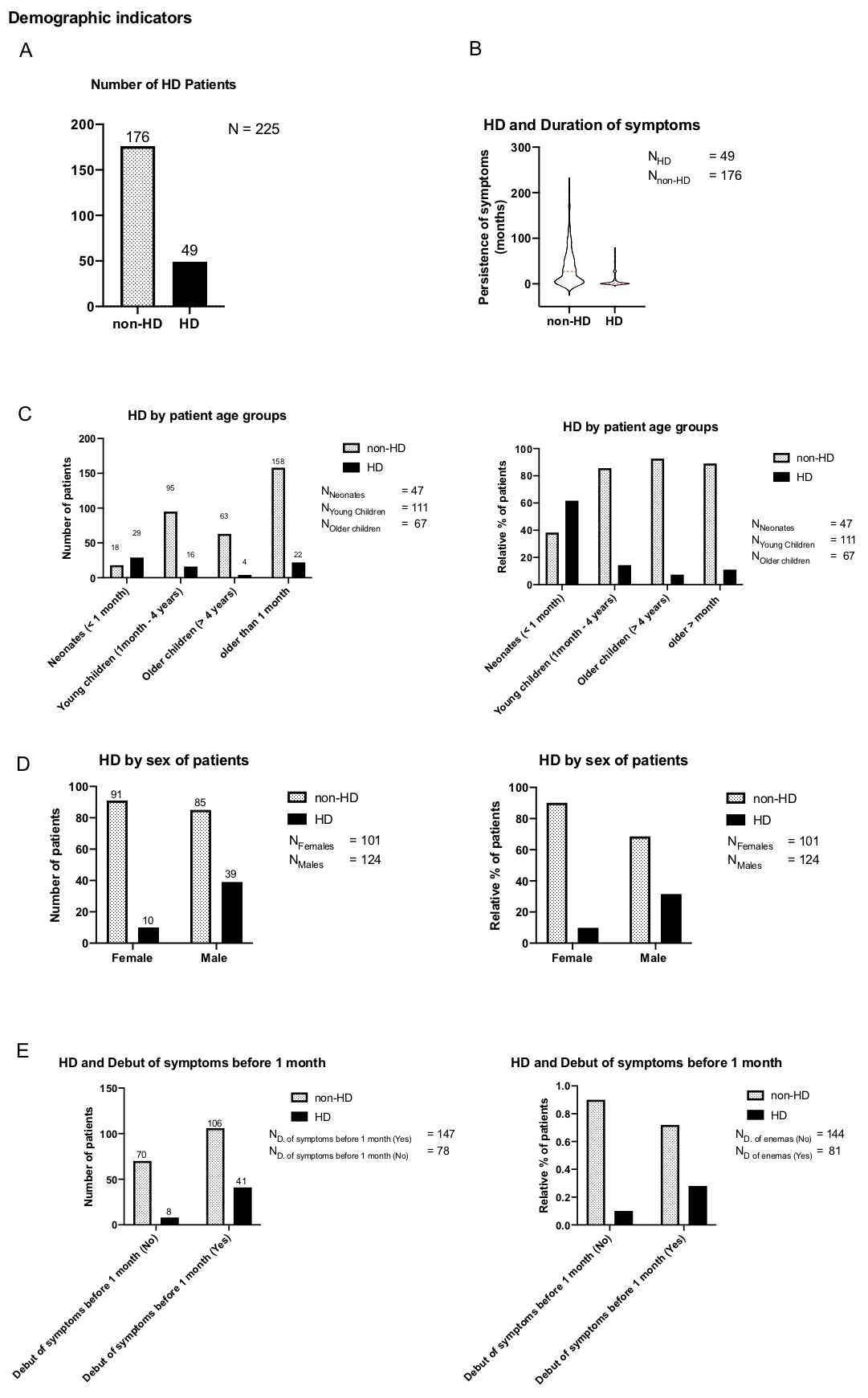

Supplement: Supplementary file 1 — Supplementary file1 (TIFF 21344 KB) [file 384_2021_4069_MOESM1_ESM.tiff]

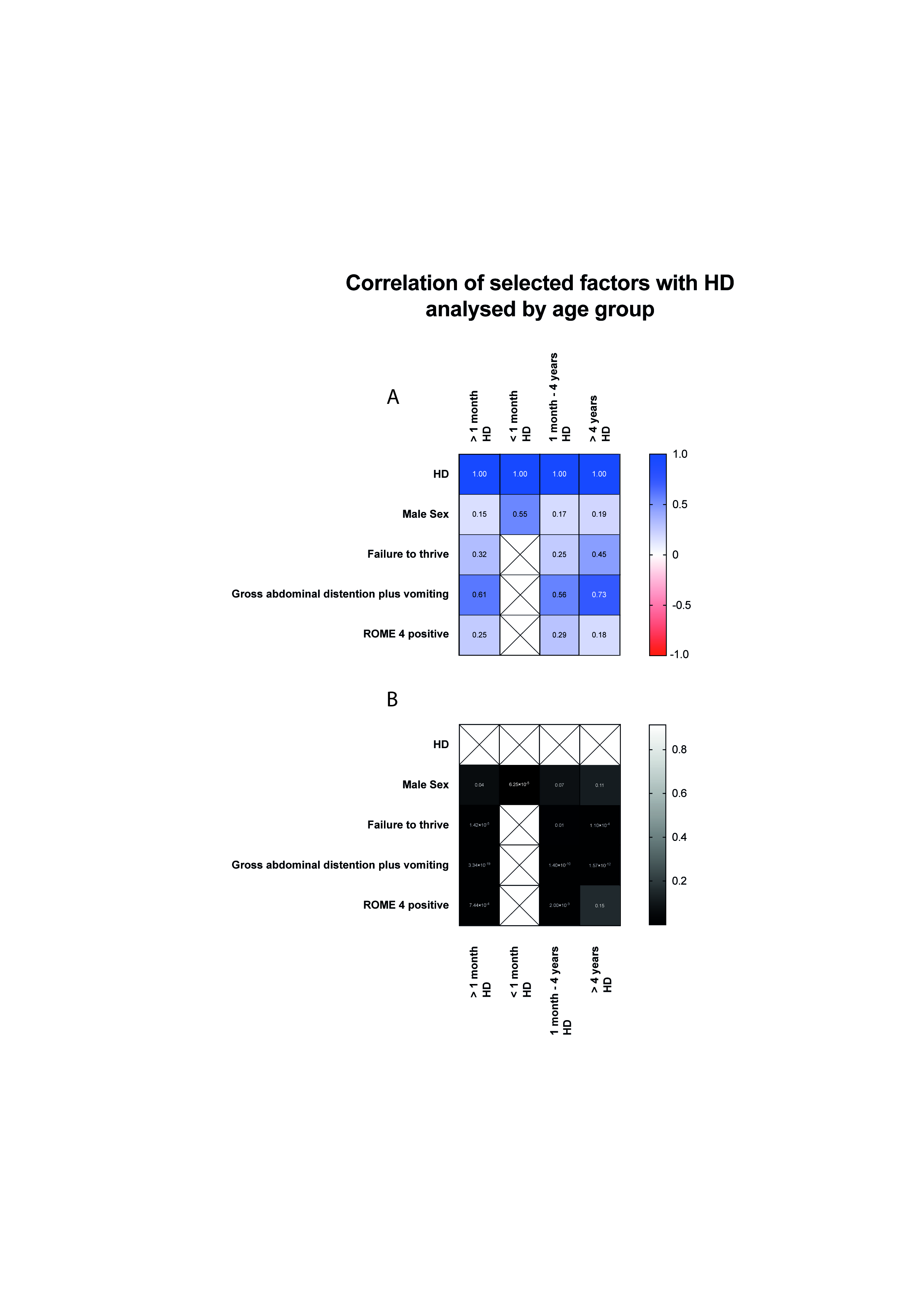

Supplement: Supplementary file 2 — Supplementary file2 (TIF 35898 KB) [file 384_2021_4069_MOESM2_ESM.tif]

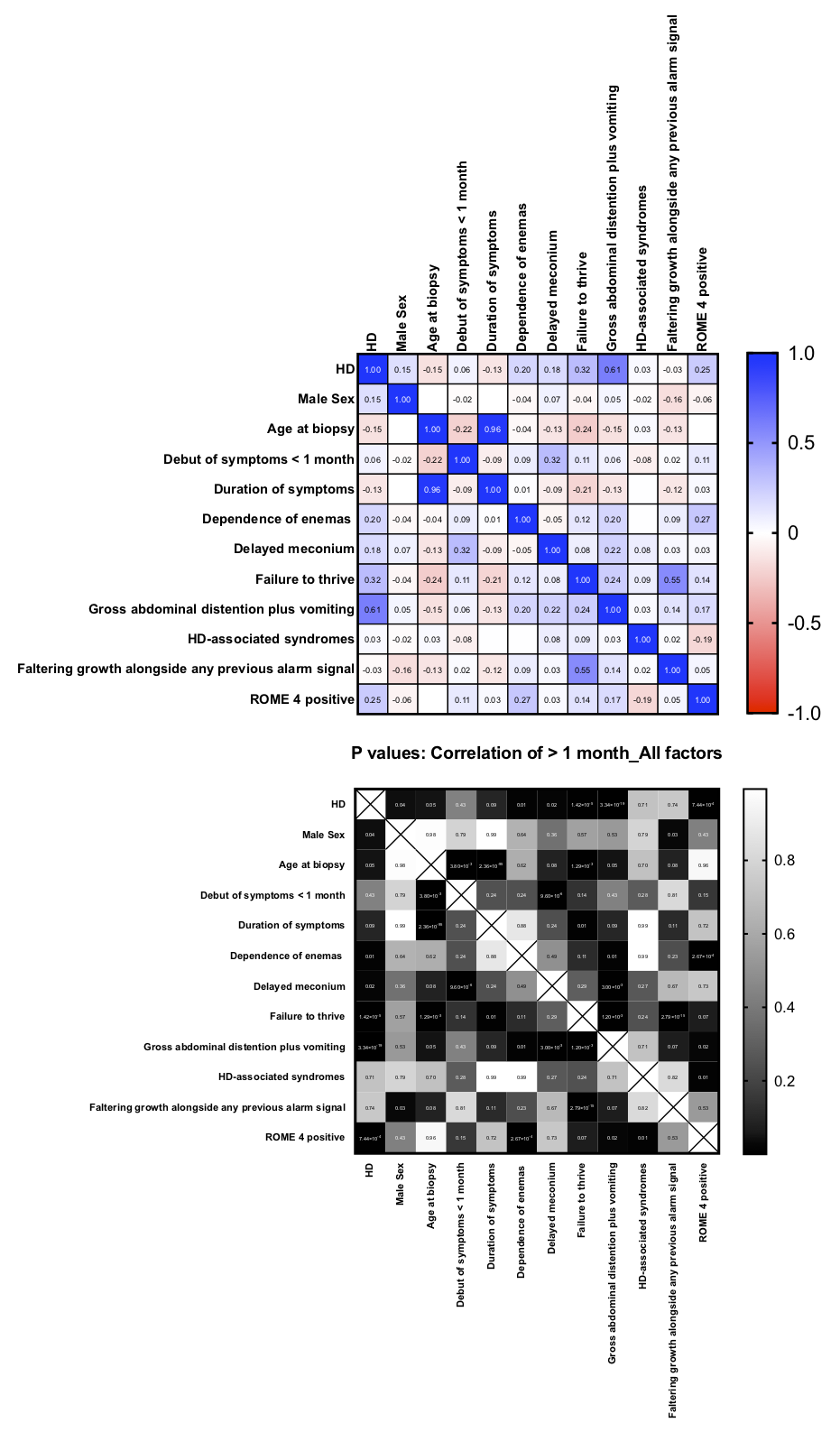

Supplement: Supplementary file 3 — Supplementary file3 (TIFF 5773 KB) [file 384_2021_4069_MOESM3_ESM.tiff]

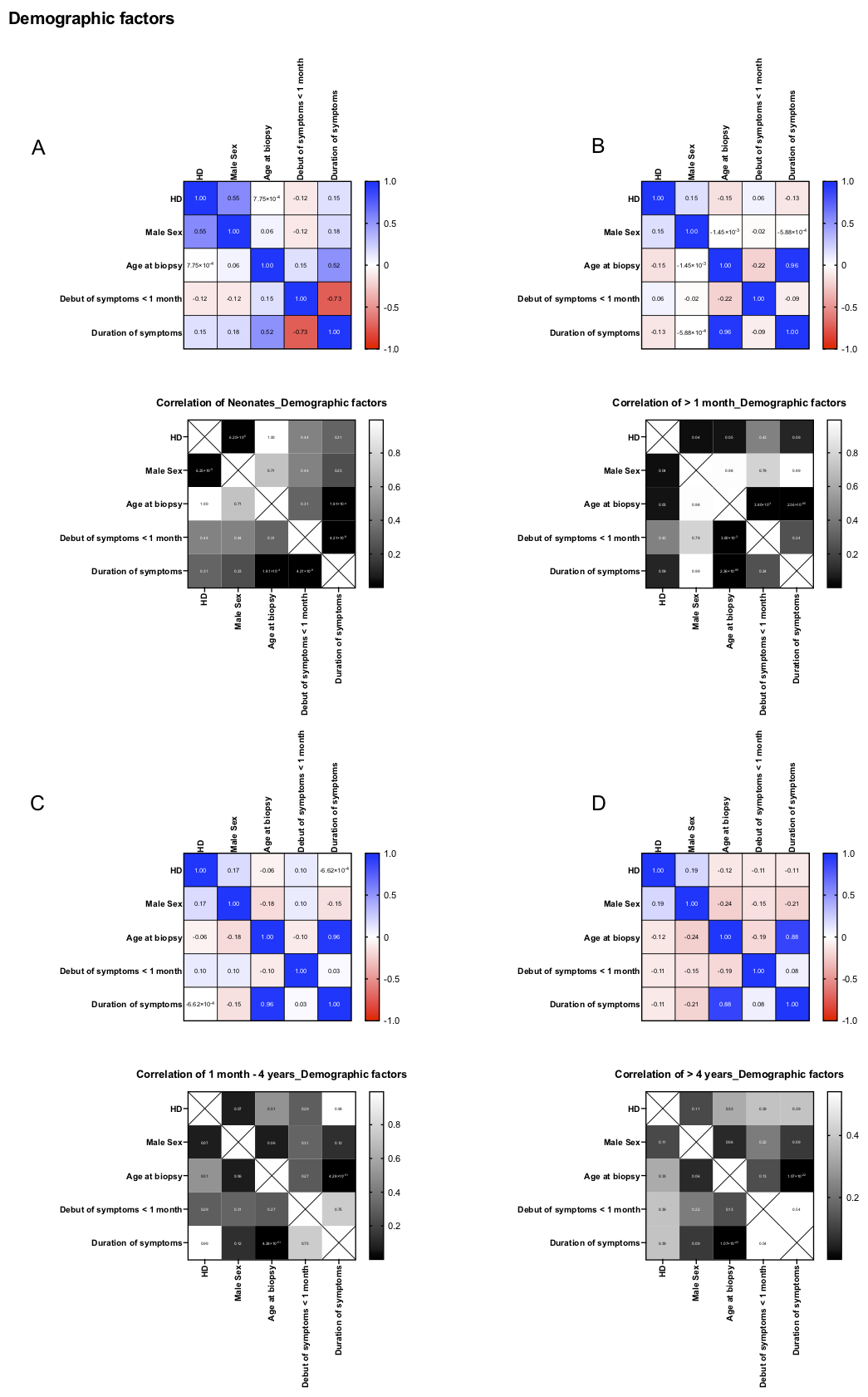

Supplement: Supplementary file 4 — Supplementary file4 (TIFF 14187 KB) [file 384_2021_4069_MOESM4_ESM.tiff]

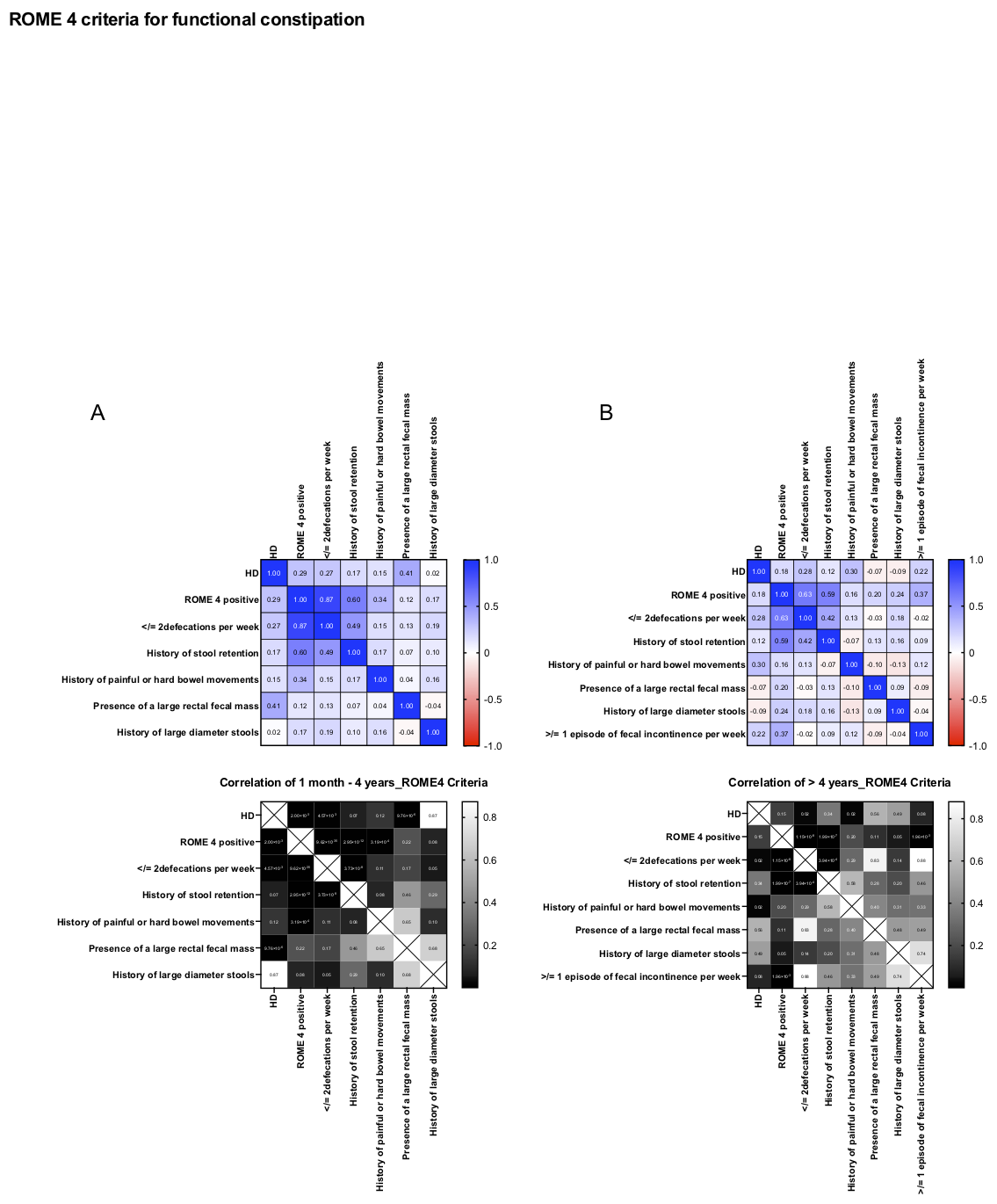

Supplement: Supplementary file 5 — Supplementary file5 (TIFF 6075 KB) [file 384_2021_4069_MOESM5_ESM.tiff]
