# Supplementary material for: Evaluation of diagnostic factors used to refer children with constipation for rectal biopsies
Source: Int J Colorectal Dis. 2021 Dec 9;37(3):597–605. doi: 10.1007/s00384-021-04069-4 (PMC8885502; doi:10.1007/s00384-021-04069-4)
Supplement: Supplementary file 6 — Supplementary file6 (PDF 84 KB) [file 384_2021_4069_MOESM6_ESM.pdf]

Table 1

Age	Total	Female	Male
**Neonates (< 1 month)**	47<br>(29 HD ≈ 62%)	18<br>(5 HD ≈ 28%)	29<br>(24 HD ≈ 83%)
**> 1 month**	178<br>(20 HD ≈ 11%)	83<br>(5 HD ≈ 6%)	95<br>(15 HD ≈ 16%)
**1 month - 4 years**	111<br>(16 HD ≈ 14%)	58<br>(5 HD ≈ 9%)	53<br>(11 HD ≈ 21%)
**> 4 years**	67<br>(4 HD ≈ 6%)	25<br>(0 HD = 0%)	42<br>(4 HD ≈ 10%)

Table 2A

Factor category	Factor	non-HD (18)	HD (29)	Correlation with HD	Confidence interval	p-value
**Demographic factor**	**Male sex**	5/18 (≈ 28%)	24/29 (≈ 83%)	0.55	0.32 to 0.72	**<u>< 0.01</u>**
**Alarm signal for HD**	**Delayed meconium**	11/18 (≈ 61%)	19/29 (≈ 65%)	0.05	-0.25 to 0.33	0.77
**Alarm signal for HD**	**Anorectal stimulation**	1/18 (≈ 6%)	4/29 (≈ 14%)	0.13	-0.16 to 0.40	0.38

Table 2B

Factor category	Factor	Odds	Standard error	95% CI (profile likelihood)	p-value
	**Intercept**	0.38	0.53	-2.09 to 0.02	0.07
**Demographic factor**	**Male sex**	12.48	0.72	1.19 to 4.04	**<u>< 0.01</u>**

Table 3A

Factor category	Factor	non-HD (158)	HD (20)	Correlation with HD	Confidence interval	p-value
ROME 4 criteria for functional constipation	ROME4 (+)	99/158 (≈ 63%)	20/20 (=100%)	0.25	0.11 to 0.38	**< 0.01**
Demographic factor	Male sex	80/158 (≈ 51%)	15/20 (≈ 75%)	15.00	0.01 to 0.29	**0.04**
Demographic factor	Age at biopsy (months)	35 (1 - 209)	15.5 (2 - 85)	-0.15	-0.29 to -0.00	**0.048**
Demographic factor	Debut of symptoms before 1 month of age	88/158 (≈ 56%)	13/20 (≈ 65%)	0.06	-0.09 to 0.20	0.43
Demographic factor	Duration of symptoms (months)	31 (0.13 - 207)	22.5 (1 - 74)	-0.13	-0.15 to 0.15	0.09
Alarm signal for HD	Dependence of enemas	62/158 (≈ 39%)	14/20 (≈ 70%)	0.20	0.05 to 0.33	**0.01**
Alarm signal for HD	Delayed meconium	33/158 (≈ 21%)	9/20 (≈ 45%)	0.18	0.03 to 0.32	**0.02**
Alarm signal for HD	Failure to thrive	38/158 (≈ 24%)	14/20 (≈ 70%)	0.32	0.18 to 0.45	**< 0.01**
Alarm signal for HD	Gross abdominal distention plus vomiting	7/158 (≈ 4%)	13/20 (≈ 65%)	0.61	0.50 to 0.69	**< 0.01**
Alarm signal for HD	HD associated syndromes	12/158 (≈ 8%)	2/20 (≈ 10%)	0.03	-0.12 to 0.17	0.71
Alarm signal for HD	Faltering growth in addition to any previous alarm signal	20/158 (≈ 13%)	2/20 (≈ 10%)	-0.03	-0.17 to 0.12	0.23

Table 3B

Factor category	Factors	Odds	Standard error	95% CI (profile likelihood)	p-value
	Intercept	0.0004152	1.971	-13.27 to -4.70	<0.01
ROME 4 criteria for functional constipation	ROME4 (+)	24.18	1.637	0.76 to 4.87	0.05
Demographic factor	Male sex	5.676	1.736	0.31 to 3.42	**0.03**
Demographic factor	Age at biopsy	0.9844	0.01279	-0.04 to 0.01	0.22
Alarm signal for HD	Dependence of enemas	2.575	0.7898	-0.57 to 2.60	0.23
Alarm signal for HD	Delayed meconium	2.058	0.8114	-0.93 to 2.33	0.37
Alarm signal for HD	Failure to thrive	5.635	0.7292	0.36 to 3.29	**0.02**
Alarm signal for HD	Gross abdominal distention plus vomiting	24.57	0.7737	1.78 to 4.87	**< 0.01**

Table S1

	non-HD	HD	Total
**All patients**	176	49	225
**Age < 1 month**	18	29	47
**Age > 1 month**	158	20	178
**Age 1 month - 4 years**	95	16	111
**Age > 4 years**	63	4	67
**ROME4 (-)**	59	0	59
**ROME4 (+)**	99	20	119
**ROME4 NA (Age < 1 month)**	18	29	47
**Female**	91	10	101
**Male**	85	39	124
**Dependence of enemas (No)**	113	31	142
**Dependence of enemas (Yes)**	63	18	81
**Delayed meconium (No)**	132	21	153
**Delayed meconium (Yes)**	44	28	72
**Associated syndromes (No)**	161	40	201
**Associated syndromes (Yes)**	15	9	24

Table S2A

Factor category	Factors	non-HD (95)	HD (16)	Correlation with HD	Confidence interval	p-value
ROME 4 criteria for functional constipation	ROME4 (+)	58/95 (≈ 61%)	16/16 (= 100%)	0.29	0.11 to 0.45	**< 0.01**
Demographic factor	Male sex	42/95 (≈ 44%)	11/16 (≈ 68%)	0.17	-0.01 to 0.35	0.07
Demographic factor	Age at biopsy (months)	12 (1 -109)	8.5 (2 - 47)	-0.06	-0.25 to 0.12	0.50
Demographic factor	Debut of symptoms before 1 month of age	58/95 (≈ 61%)	12/16 (≈ 75%)	0.10	-0.08 to 0.28	0.23
Demographic factor	Duration of symptoms (months)	10 (0.13 - 97)	9.5 (1- 47)	-6.62e-004	-0.19 to 0.19	0.99
Alarm signal for HD	Dependence of enemas	36/95 (≈ 38%)	13/16 (≈ 81%)	0.31	0.13 to 0.46	**< 0.01**
Alarm signal for HD	Delayed meconium	21/95 (≈ 22%)	7/16 (≈ 44%)	0.18	-0.01 to 0.35	0.07
Alarm signal for HD	Failure to thrive	32/95 (≈ 34%)	11/16 (≈ 69%)	0.25	0.07 to 0.42	**< 0.01**
Alarm signal for HD	Gross abdominal distention plus vomiting	6/95 (≈ 6%)	10/16 (≈ 62%)	0.56	0.42 to 0.68	**< 0.01**
Alarm signal for HD	HD associated syndromes	6/95 (≈ 6%)	2/16 (≈ 12.5%)	0.08	-0.10 to 0.27	0.38
Alarm signal for HD	Faltering growth in addition to any previous alarm signal	17/95 (≈ 18%)	2/16 (≈ 12.5%)	-0.05	-0.23 to 0.14	0.60

Table S2B

Factor category	Factors	Odds	Standard error	95% CI (profile likelihood)	p-value
	Intercept	0.002093	1.78	-11.43 to -3.54	< 0.01
ROME 4 criteria for functional constipation	ROME4 (+)	17.84	1.65	0.39 to 8.00	0.08
Alarm signal for HD	Dependence of enemas	2.88	0.77	-0.41 to 2.70	0.17
Alarm signal for HD	Failure to thrive	17.87	0.71	1.50 to 4.45	0.17
Alarm signal for HD	Gross abdominal distention plus vomiting	17.84	0.74	0.39 to 8.00	**< 0.01**

Table S3A

Factor category	Factor	non-HD (63)	HD (4)	Correlation with HD	Confidence interval	p-value
ROME 4 criteria for functional constipation	ROME4 (+)	41/63 (≈ 65%)	4/4 (=100%)	0.18	0.07 to 0.40	0.15
Demographic factor	Male sex	38/63 (≈ 60%)	4/4 (=100%)	0.19	0.05 to 0.42	0.12
Demographic factor	Age at biopsy (months)	83 (48 - 209)	74,5 (61 -85)	-0.12	-0.35-0.12	0.05
Demographic factor	Debut of symptoms before 1 month of age	30/63 (≈ 48%)	1/4 (= 25%)	-0.11	-0.34 to 0.14	0.22
Demographic factor	Duration of symptoms (months)	70 (24 -207)	64.5 (51 - 74)	-0.11	-0.34 to 0.14	0.09
Alarm signal for HD	Dependence of enemas	26/63 (≈ 41%)	1/4 (= 25%)	-0.08	-0.31 to 0.17	0.53
Alarm signal for HD	Delayed meconium	12/63 (≈ 19%)	2/4 (= 50%)	0.18	0.06 to 0.40	0.14
Alarm signal for HD	Failure to thrive	6/63 (≈ 10%)	3/4 (= 75%)	0.46	0.24 to 0.63	**< 0.01**
Alarm signal for HD	Gross abdominal distention plus vomiting	1/63 (≈ 2%)	3/4 (= 75%)	0.73	0.60 to 0.83	**< 0.01**
Alarm signal for HD	HD associated syndromes	6/63 (≈ 10%)	0/4 (= 0%)	-0.08	-0.31 to 0.16	0.53
Alarm signal for HD	Faltering growth in addition to any previous alarm signal	3/63 (≈ 5%)	0/4 (= 0%)	0.06	-0.29 to 0.18	0.66
Alarm signal for HD	Fecal incontinence	20/63 (≈ 32%)	3/4 (= 75%)	0.22	-0.03 to 0.44	0.08

Table S4A

Factors	non-HD (158)	HD (20)	Correlation with HD	Confidence interval	p-value
**ROME 4 (+)**	99/158 (≈ 63%)	20/20 (= 100%)	0.25	0.11 to 0.38	< 0.01
2 or fewer defecations per week	80/158 (≈ 51%)	19/20 (= 95%)	0.28	0.14 to 0.41	**< 0.01**
History of stool retention	129/158 (≈ 82%)	20/20 (=100%)	0.16	0.01to 0.30	**0.04**
History of painful or hard bowel movements	25/158 (≈16%)	5/20 (= 25%)	0.20	0.05 to 0.33	**0.01**
Presence of a large fecal mass in the rectum	5/158 (≈ 3%)	3/20 (= 15%)	0.18	0.03 to 0.32	**0.02**
History of large diameter stools	12/158 (≈ 8%)	1/20 (= 5%)	-0.03	-0.18 to 0.11	0.68

Table S4B

Factors	Odds	Standard error	95% CI (profile likelihood)	p-value
**Intercept**	< 0.01	1.60	4.42 e-005 to 0.07	< 0.01
2 or fewer defecations in the toilet per week	11.28	1.00	2.23 to 142,30	**< 0.01**
History of stool retentive posturing or excessive volitional stool retention	1.66	1.76	0.09 to 330.20	0.77
History of painful or hard bowel movements	3.12	0.54	1.06 to 9.07	**0.04**
Presence of a large fecal mass in the rectum	8.68	0.89	1.45 to 54.81	**0.01**

Table S5A

Factors	non-HD (95)	HD (16)	Correlation with HD	Confidence interval	p-value
**ROME 4 (+)**	58/95 (≈ 61%)	16/16 (=100%)	0.29	0.11 to 0.45	< 0.01
2 or fewer defecations in the toilet per week	54/95 (≈ 57%)	15/16 (≈ 94%)	0.27	0.09 to 0.43	**< 0.01**
History of stool retentive posturing or excessive volitional stool retention	78/95 (≈ 82%)	16/16 (=100%)	0.17	-0.01 to 0.35	0.07
History of painful or hard bowel movements	19/95 (≈ 20%)	6/16 (≈ 38%)	0.15	-0.04 to 0.32	0.12
Presence of a large fecal mass in the rectum	0/95 (≈ 0%)	3/16 (≈ 19%)	0.41	0.24 to 0.55	**< 0.01**
History of large diameter stools that may obstruct the toilet	5/95 (≈ 5%)	1/16 (≈ 6%)	0.02	-0.17 to 0.20	0.87

Table S5B

Factors	Odds	Standard error	95% CI (profile likelihood)	p-value
**Intercept**	0.02	1.02	0.001 to 0.11	< 0.01
2 or fewer defecations in the toilet per week	9.57	1.07	1.76 to 179.2	**0.03**
Presence of a large fecal mass in the rectum	282.60	2.56	6.83 to 1881103	**0.03**

Table S6A

Factors	non-HD (63)	HD (4)	Correlation with HD	Confidence interval	p-value
**ROME 4 (+)**	41/63 (≈ 65%)	4/4 (= %)	0.18	-0.07 to 0.40	0.15
2 or fewer defecations in the toilet per week	26/63 (≈ 41%)	4/4 (=100%)	0.28	0.04 to 0.49	**0.02**
History of stool retentive posturing or excessive volitional stool retention	51/63 (≈ 80%)	4/4 (=100%)	0.12	-0.13 to 0.35	0.34
History of painful or hard bowel movements	6/63 (≈ 10%)	2/4 (= 50%)	0.30	0.06 to 0.50	**0.02**
Presence of a large fecal mass in the rectum	5/63 (≈ 8%)	0/4 (= 0%)	-0.07	-0.30 to 0.17	0.57
History of large diameter stools that may obstruct the toilet	7/63 (≈ 11%)	0/4 (= 0%)	-0.09	-0.32 to 0.16	0.49
At least 1 episode of fecal incontinence per week	20/63 (≈ 32%)	3/4 (= 75%)	0.22	-0.03 to 0.44	0.08
